# Construction of an Electrochemical Impedance Spectroscopy Matching Method Based on Adaptive Multi-Error Driving and Application Testing for Biofilm Impedance Verification

**DOI:** 10.3390/bios15090604

**Published:** 2025-09-12

**Authors:** Hanyang Bao, Fan Yu, Peiyan Dai, Boyu Guo, Ying Xu

**Affiliations:** 1 School of Hangzhou Dianzi University ITMO Joint Institute, Hangzhou Dianzi University, Hangzhou 310027, China; 2 Provincial Key Laboratory of Soft Matter & Biomedical Materials, Wenzhou Institute of the University of Chinese Academy of Sciences (WIUCAS), Wenzhou 325000, China

**Keywords:** electrochemical impedance spectroscopy, dataset decomposition, error adaptive feedback, DE-LM parameter optimization

## Abstract

Electrochemical impedance spectroscopy (EIS) is a technique used to analyze the kinetics and interfacial processes of electrochemical systems. The selection of an appropriate equivalent circuit model for EIS interpretation was traditionally reliant on expert experience, rendering the process subjective and prone to error. To address these limitations, an automated framework for both model selection and parameter estimation was proposed. The methodology was structured such that initial model screening was performed by a global heuristic search algorithm, adaptive optimization was guided by an integrated XGBoost-based error feedback mechanism, and precise parameter estimation was achieved using a Differential Evolution–Levenberg–Marquardt (DE-LM) algorithm. When evaluated on a purpose-built dataset comprising 4.8 × 10^5^ spectra across diverse circuit and biofilm scenarios, a model classification accuracy of 96.32% was achieved, and a 72.3% reduction in parameter estimation error was recorded. The practical utility of the method was validated through the quantitative analysis of bovine serum albumin–Clenbuterol hydrochloride (BSA-CLB), wherein an accuracy of 95.2% was demonstrated and a strong linear correlation with target concentration (R^2^ = 0.999) was found. Through this approach, the limitations of traditional black-box models were mitigated by resolving the physical meaning of parameters. Consequently, the automated and quantitative monitoring of processes such as biofilm formation was facilitated, enabling the efficient evaluation of antimicrobial drugs or anti-fouling coatings.

## 1. Introduction

Electrochemical impedance spectroscopy (EIS) is a non-destructive diagnostic that resolves the kinetic and interfacial processes of electrochemical systems in the frequency domain and has been widely adopted in energy storage, corrosion monitoring, and bio-sensing [1,2].

The equivalent-circuit model (ECM) expresses processes of charge transfer, diffusion, and double-layer phenomena through combinations of circuit elements. By fitting these elements, it provides quantitative parameters that greatly improve interpretability for complex systems [3,4]. However, manual circuit selection is subjective, sensitive to initial guesses, and easily trapped in local minima, which limits the accuracy and reproducibility of the analysis [5].

To overcome these limitations, data-driven methods like machine learning are increasingly used to interpret EIS [6,7]. While these models show success in specific tasks such as battery diagnostics and biosensing [8,9,10,11], they often face two critical limitations. First, most are trained on single-type datasets, which restricts their generalizability across different electrochemical systems [12]. Second, many function as ‘black boxes’, failing to provide the clear physicochemical insights offered by traditional ECMs [9,13].

Here, a global heuristic framework is proposed for the selection and calibration of intelligent ECMs based on heterogeneous EIS data, with a view to addressing this issue. While earlier studies have effectively employed machine learning for EIS analysis, their approaches often relied on pre-selected ECMs. For instance, C. Wang et al. [10] used pre-defined ECMs for quantitative sensing, and Q.-K. Wang et al. [14] modeled the complex dependencies of specific ECM parameters on system state variables. Therefore, the holistic framework introduced in this study enables full process automation. The characteristic of this framework is that it incorporates four major innovations: firstly, an adaptive multi-criteria evaluation engine based on XGBoost is introduced to enable intelligent selection of robust data-driven models; secondly, a self-correcting DE-LM optimization algorithm with an automatic restart strategy is developed to ensure global parameter convergence; thirdly, a high-throughput parallel computing architecture is constructed to significantly enhance the efficiency of large-scale data processing. Most notably, physical constraints are deeply embedded throughout the entire pipeline to consistently preserve physical consistency and model interpretability.

By integrating these components, we advance beyond empirical fitting and black-box prediction limitations. By integrating a multi-model optimization strategy with physics-informed error metrics, we advance beyond empirical fitting and black-box prediction limitations. The ultimate goal is to deliver an automated, reliable and interpretable impedance analytics tool for complex electrochemical systems.

## 2. Materials and Methods

**Figure 1:**
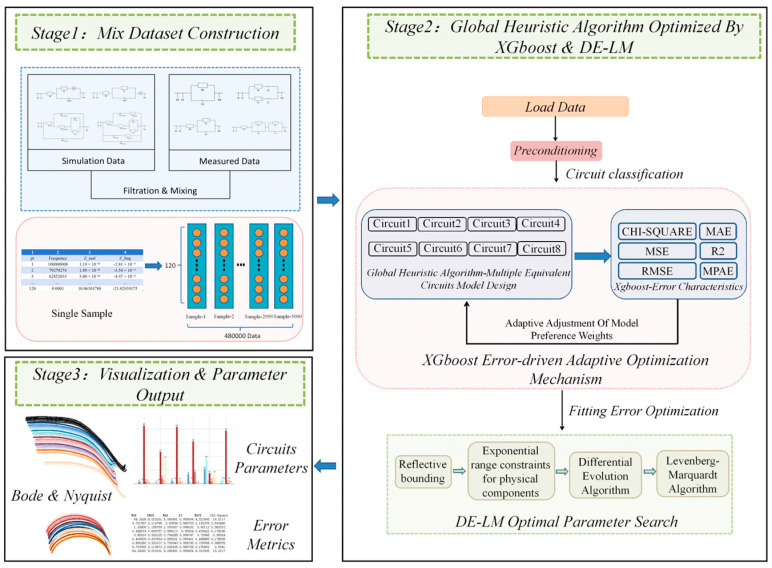
Workflow of the global heuristic algorithm for EIS fitting, incorporating multi-model parallel optimization and an error-driven adaptive mechanism.

The global heuristic fitting algorithm optimized for EIS based on XGBoost consists of three parts, as illustrated in Figure 1. Initially, experimental data was collected with a portable electrochemical workstation developed by our research team. The experimental data was generated using custom-synthesized, PEG-functionalized Fe_3_O_4_@SiO_2_ core–shell magnetic nanoparticles, which were applied to a magnetic glassy carbon electrode to form sensing layers by adsorbing bovine serum albumin–clenbuterol (BSA-CLB) (Luo Yang, China)from solutions of varying concentrations. The EIS measurements were conducted in a 20 mM [Fe(CN)_6_]^3−^/^4−^ solution using a standard three-electrode system, sweeping from 10 kHz down to 10 Hz at open circuit potential. Each measurement was repeated 10 times to ensure statistical robustness.

Simulated data is generated following a series of meticulous filtration procedures, including noise elimination, anomaly detection, and frequency band calibration, a set of systematic procedures is initiated. In Section S1.1 of the SI we concisely describe the methodology for generating simulation data. These procedures include feature alignment and enhanced sampling, culminating in the generation of a structured dataset comprising 4000 authentic RC circuit hybrid biofilm samples and 480,000 records. The data features encompass multi-dimensional information, including the real and imaginary parts of impedance and frequency. A multi-dimensional validation system, incorporating the Kramers–Kronig transformation and time constant distribution analysis, is employed to ensure that the data is both physically reasonable and computationally viable.

The Stage 2 in Figure 1 presents a flowchart illustrating the globally heuristic algorithm, which is designed to solve the dual-objective problem of accurate model selection and high-fidelity parameter estimation. The fitting process first performs a feature importance analysis on multiple error metrics using XGBoost. This analysis is conducted dynamically [15], with model weights adjusted accordingly to achieve an error-driven adaptive optimization for circuit classification. This initial stage is critical, as it correctly identifies the most probable equivalent circuit model for a given EIS spectrum. The detailed mechanism of this error-adaptive framework, which forms the basis of our approach, is elaborated in Section 2.2.1.

After the optimal circuit is classified, the algorithm proceeds to the parameter estimation phase. To enhance fitting accuracy, an optimal parameter space search is essential, as default initial parameters often lead to suboptimal results. In the preprocessing step, reflective boundary constraints and physical component index range constraints are applied. Subsequently, a hybrid global-local search is performed, beginning with a differential evolution algorithm for robust global exploration, followed by local refinement optimization using the Levenberg–Marquardt algorithm to ensure precision. This synergistic process, which will help to reduce parameter estimation error, completes the classification of the optimal circuit and the optimization of fitting accuracy. The synergistic design and validation of the DE-LM parameter optimization approach are further detailed in Section 2.2.2. Additionally, the system supports parallel fitting of eight circuit models (Circuit-1 to 8), including the classical *Randles* model and bio-membrane-modified electrode configurations.

In the final stage of the experiment, a multi-dimensional electrochemical impedance analysis visualization and parameter output system was established. The validity of the system’s high-fidelity results was verified using dual-mode visualization techniques (including Nyquist plots and Bode plots). The parameter output module systematically extracts key component parameters of the R-CPE-W composite circuit based on equivalent circuit model inversion and employs thermodynamic constraint verification (Kramers–Kronig transformation residual < 0.1%). The error analysis module integrates six core metrics: chi-square test value (χ^2^), mean absolute error (MAE), mean squared error (MSE), coefficient of determination (R^2^), root mean squared error (RMSE), and mean absolute percentage error (MAPE). Through parameter interference resistance visualization technology, parameter sensitivity distributions can be revealed.

### 2.1. Impedance Data Construction

#### 2.1.1. Data Acquisition and Data Set Composition

The experimental EIS data were acquired using a custom-built electrochemical workstation, with a foundational dataset originating from experiments on biofilm systems. To significantly expand this dataset for robust model training, it was augmented with a large volume of simulated data. The generation of this simulated data was specifically designed to mimic the electrochemical evolution of biofilms [16]. This was achieved by systematically varying the parameters of relevant equivalent circuits—including those with Warburg elements and additional R-C modules—within ranges that are physically representative of key biofilm processes. These processes include changes in charge transfer resistance due to surface coverage, variations in mass diffusion limitations corresponding to film thickness and porosity, and the evolving intrinsic resistive and capacitive properties of the film itself.

The data acquisition process for both experimental and simulated data involved systematically sampling the electrochemical impedance at 120 frequency points, spanning a range from 0.001 Hz to 10^5^ Hz. As detailed in Supplementary Information Figure S1, each data point at a specific frequency (F_req_) contains four attributes: an index (Pt), the frequency, and the impedance’s real (Z_real_) and imaginary (Z_imag_) parts. Consequently, a complete impedance spectrum constitutes a single sample with 480 feature attributes. To enhance the model’s generalization capabilities, experimental data were combined with the biofilm-centric simulated data, creating a final dataset of 4000 samples. This methodology introduces multi-scenario impedance features, yielding an EIS dataset characterized by broad frequency coverage, a rich distribution of dynamic parameters, and strict physical consistency. It is ideally suited for accurately characterizing multi-scale electrochemical responses, from interfacial relaxation processes to diffusion kinetics.

#### 2.1.2. Equivalent Circuit Model Selection

As detailed in the reference library of ECMs in Figure S2a, our analytical framework is built upon a progression of established electrochemical representations [17]. The cornerstone is the classical *Randles* circuit, which describes a simple, kinetically controlled interface by combining a solution resistance (R_s_) with a parallel charge transfer resistance (R_ct_) and a double-layer capacitance (C_dl_). The R_ct_, visualized as the semicircle diameter in the Nyquist plot, directly quantifies the opposition to the flow of electrons during a redox reaction, thus providing a direct measure of the reaction kinetics. For systems where the reaction rate is limited not by kinetics but by the physical transport of reactants to the electrode, the model is necessarily extended to include a Warburg element (W). This diffusive element accounts for semi-infinite linear diffusion and manifests as a characteristic 45° tail in the low-frequency region of the EIS spectrum, allowing for the separation of kinetic and mass-transport control regimes [18].

Following this established logic, a specific classification tree (Figure S2b) was constructed to deconstruct the impedance data of our system by systematically comparing three key models. First, the Bare Electrode Model provides an essential baseline by characterizing the unmodified interface. It employs a Constant Phase Element (CPE) in place of a pure capacitor to more accurately represent the non-ideal, frequency-dependent capacitive behavior that arises from the microscopic roughness, porosity, and chemical heterogeneity inherent to a real-world electrode surface. This model is critical for isolating the intrinsic charge transfer properties of the bare electrode before any modification. Second, the specialized Magnetic-based Nano-Biofilm Model was developed by incorporating a Warburg element (W) into the bare electrode framework. The addition of this element is physically justified and necessary because the assembled nanoparticle film, while porous, creates a tortuous path for electroactive species. The Warburg element’s function is therefore to quantitatively model this mass diffusion limitation, effectively describing the increased difficulty for ions to travel through the nanoparticle layer to reach the active electrode surface. Finally, the Generalized Biofilm Model offers a more phenomenological, layered perspective. Its unique contribution is the addition of a distinct parallel R-C element (R_biofilm_ || C_biofilm_). This addition is crucial for systems where the film itself has significant bulk electrical properties. This allows for the de-convolution of the film’s intrinsic ionic resistance (R_biofilm_) and dielectric capacitance (C_biofilm_) from the separate charge transfer processes (R_ct_ and CPE_dl_) occurring at the underlying electrode-film interface. This systematic comparison provides a systematic theoretical foundation for assigning physical meaning to impedance parameters in complex systems like biosensors.

### 2.2. Optimization Method Based on Global Heuristic Algorithm

#### 2.2.1. An Error-Driven Adaptive Optimization Approach

**Figure 2:**
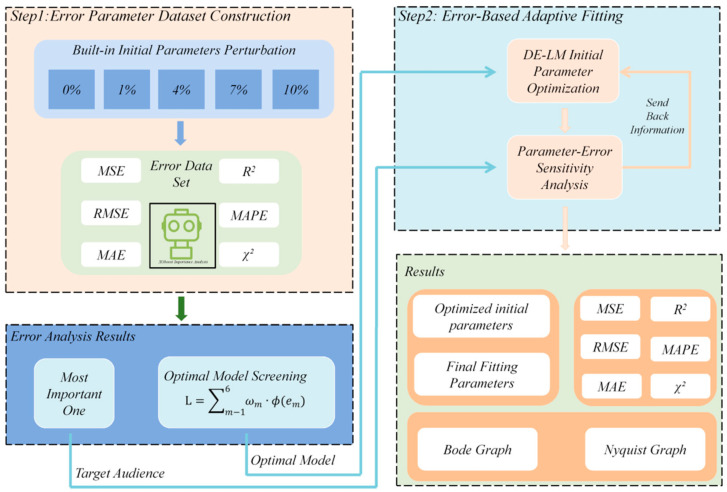
Error-driven adaptive optimization strategy.

After constructing a vigorously validated dataset encompassing diverse scenarios relevant to biofilm electrochemical analysis, the classification of the equivalent circuit model can be approached using traditional global heuristic algorithms based on a single fitting error. However, this traditional single-objective optimization is easily limited by the one-sidedness of any single error metric. To overcome this, the thesis provides a comprehensive overview of model performance by integrating six complementary metrics, as shown in the flowchart in Figure 2. The χ^2^ test ensures distributional consistency, capturing subtle deviations in the low-frequency diffusion region. MAE and MAPE enhance robustness against high-frequency noise, preventing outliers from dominating the optimization. Synergistically, R^2^ and RMSE evaluate the fidelity of global trend fitting, balancing both amplitude and phase error contributions.

Based on this multi-metric foundation, we sought to identify the most effective machine learning algorithm to drive our adaptive optimization framework. The Boost family of algorithms was identified as a strong candidate due to its ability to handle complex, non-linear interactions and provide quantitative feature importance metrics, which are crucial for our error-feedback mechanism [19,20,21].

To ensure the selection of the optimal algorithm, we conducted a systematic comparative analysis of three prominent Boost implementations: AdaBoost, CatBoost, and XGBoost. The goal was to empirically determine which algorithm offered the highest accuracy for the circuit classification task on our dataset.

Later on, we will clearly present the comparison results to demonstrate that the XGBoost-based method exhibits superior performance. XGBoost achieved a model classification accuracy of 96.32%, which represents a significant improvement over both CatBoost (87.54%) and the next-best traditional method, AdaBoost (85.5%). This superior performance is primarily attributed to XGBoost’s use of a regularized loss function (combining L1 and L2 regularization) and its optimization of gradients through second-derivative approximation, leading to enhanced generalization ability and model stability [22].

Therefore, based on this deliberate and empirically validated selection process, XGBoost was chosen as the core engine for our error-driven adaptive optimization approach. This choice was not arbitrary but rather the outcome of a rigorous evaluation aimed at maximizing the framework’s robustness and accuracy. A feedback loop was then implemented: a sensitivity analysis of the initial parameters—identifying their contribution to dominant errors—is fed back into the global heuristic algorithm for tight coupling. By applying a weighted distribution, critical parameter sensitivities are identified, enabling adaptive weight optimization to steer the search along the Pareto optimal boundary.

Each error characteristic contains χ^2^, MSE, RMSE, MAE, R^2^, MAPE, all have specific formulas and meanings, the specific formulas are shown in Equation (1).

(1) $$\left\{\begin{array}{c} CHI-SQUARE=\sum_{i=1}^{n}\frac{{\left(y_{i}-{\hat{y}}_{i}\right)}^{2}}{{\hat{y}}_{i}}\\ MSE=\frac{1}{n}\sum_{i=1}^{n}{\left(y_{i}-{\hat{y}}_{i}\right)}^{2}\\ RMSE=\sqrt{\frac{1}{n}\sum_{i=1}^{n}{\left(y_{i}-{\hat{y}}_{i}\right)}^{2}}\\ MAE=\frac{1}{n}\sum_{i=1}^{n}\left|y_{i}-{\hat{y}}_{i}\right|\\ R^{2}=1-\frac{\sum_{i=1}^{n}{\left(y_{i}-{\hat{y}}_{i}\right)}^{2}}{\sum_{i=1}^{n}{\left(y_{i}-{\bar{y}}_{i}\right)}^{2}}\\ MAPE=\frac{100\%}{n}\sum_{i=1}^{n}\left|\frac{y_{i}-{\hat{y}}_{i}}{y_{i}}\right| \end{array}\right.,$$

The χ^2^ statistic quantifies the deviation between observed and expected values in a dataset. MSE reflects the degree of deviation of the predicted value from the real value, RMSE is the square root of MSE and the scale is the same as that of the original data, MAE has low sensitivity to the outliers, R^2^ indicates the degree of model fit, and MAPE shows the proportion of deviation of the predicted value relative to the real value in percentage.

XGBoost algorithm forms a coupling mechanism with heuristic search. The decision weights of six types of indicators, MSE, RMSE, MAE, R^2^, MAPE and χ^2^, are systematically and dynamically quantified through feature importance analysis to construct asymmetric Equation (2) loss functions:

(2) $$L=\sum_{m-1}^{6}\omega_{m}\cdot \phi (e_{m}),$$

In the context of machine learning algorithms, the feature importance coefficient of XGBoost output, denoted by *ω_m_*, and the normalization function of the error indicator, represented by *ϕ*(*e_m_*), play a pivotal role in determining the efficacy of the model. This dynamic weighting strategy mitigates the limitations of the traditional fixed-weight model. It provides feedback to regulate the search direction of the global algorithm and ultimately approximate the optimal classification. Additionally, error indicators whose importance is lower than the threshold (*δ* = 0.1) are downweighted, which ensures that computational resources are clustered toward the key parameter dimensions.

#### 2.2.2. Parameter Optimization Under the DE-LM Synergistic Approach

Upon completion of the classification, the optimal circuit model for a given EIS dataset is identified. However, heuristic algorithms are often highly dependent on initial parameter settings, where inappropriate starting points can severely compromise the fitting results [23,24,25]. To address this critical challenge, a hybrid global-local optimization strategy, the DE-LM algorithm, is employed.

The distinct advantage of this approach lies in its synergistic design: DE performs a robust, global exploration of the non-convex parameter space to avoid local minima, while the LM algorithm provides rapid, fine-grained local convergence once a promising region is found. Compared to using DE or LM individually, the DE-LM algorithm not only achieves superior robustness and parameter similarity, but also delivers a remarkable reduction in fitting error. This combination of global robustness and local efficiency provides a powerful and reliable solution for the accurate parameterization of complex electrochemical equivalent circuits.

The DE-LM algorithm begins with the DE component, which is centered on the variational operator v_i_ = θr_1_ + F × (θr_2_ − θr_3_) (where F ∈ [0.4, 1.0]). Combined with a high crossover rate (CR = 0.9) to preserve population diversity, this stage performs a coarse-grained global search within a pre-determined parameter space [θ_min_, θ_max_]. This search is initiated by generating 200 to 500 initial population individuals and employing the residual sum of squares as the fitness function to filter for a Pareto frontier solution set, using a convergence threshold of ϵ = 0.1.

On this basis, the top 10% of high-adaptation individual output from DE are used as the initial guesses for the LM algorithm. The local quadratic convergence property of the LM algorithm is then utilized to achieve fine optimization of the parameters. The LM algorithm is dynamically adjusted by a damping factor μ (adaptively adjusted from 10^−3^ to 10^2^ based on Nielsen’s criterion) so that the estimation error of the equivalent circuit parameters is stabilized within the 95% confidence interval. In essence, this local optimization stage rapidly converges within the promising, locally convex domain identified by the global search, with a computational complexity of O(n^3^), where n is the parameter dimensionality.

**Figure 3:**
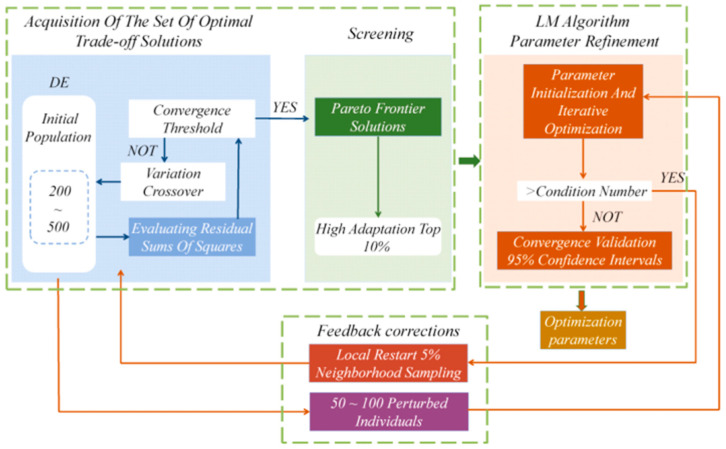
Multi-scale optimization framework combining LM with global heuristic algorithms.

To ensure robustness, if a pathological condition number (k > 10) is detected in the Jacobian matrix J, the system automatically triggers a feedback correction mechanism. This involves initiating a DE local restart strategy within a ±5% neighborhood of the current optimal solution, generating 50 to 100 perturbed individuals through resampling to reinitialize the LM iteration. This iterative validation mechanism allows the algorithm to circumvent local minima traps, effectively overcoming the effect of data dispersion on the Jacobian matrix, particularly in the high-frequency region.

(3) $${min}_{\theta }\sum_{i-1}^{N}{\left[Z_{real,i}-f_{real}(\omega_{i};\theta )\right]}^{2}+{\left[Z_{imag,i}-f_{imag}(\omega_{i};\theta )\right]}^{2},$$

where $Z_{real,i}$ and $Z_{imag,i}$ are the measured values of the real and imaginary parts of the impedance at the *i* frequency point *ω_i_*, respectively, and $f_{real}$ and $f_{imag}$ are the analytical expressions of the equivalent circuit model. The Levenberg–Marquardt (LM) algorithm is used to realize the solution of Equation (4) with the iterative equation:

(4) $$(J^{T}J+\mu I)\Delta \theta =J^{T}r,$$

where *J* ∈ R^2*N*×*n*^ is the Jacobi matrix of the residual vector *r* = [*Z*_real_ − *f*_real_, *Z*_imag_ − *f*_imag_]*^T^* and µ is the dynamic damping factor. This parameter search algorithm works in concert as Figure 3 shown below.

In the context of extensive EIS data analysis, encompassing numerous volumes, global heuristic algorithms that have been optimized with error adaptive feedback, in conjunction with DE-LM parametric algorithms, still will encounter computational efficiency limitations. These limitations arise from their nonlinear characteristics and the stringent high-dimensional optimization requirements they necessitate. In scenarios involving multiple files and models, the conventional serial fitting method exhibits a linear relationship between computation time and task size, thereby impeding the efficacy of high-throughput data analysis. The present study proposes an optimization strategy based on multilevel parallel architecture, which has been demonstrated to significantly improve the scientific research efficiency and engineering practicability of complex EIS data processing. This improvement is achieved through the implementation of algorithmic reconfiguration and dynamic scheduling of computational resources. The flowchart is displayed in Figure S2.

The fundamental technical innovation is a hierarchical parallelization framework designed to drastically reduce processing time. At its core, the framework employs thread-level parallelism to simultaneously fit multiple circuit topologies while a dedicated thread pool manages concurrent file I/O operations. The overall flowchart is shown in Figure S3. This dual-layer parallelization is orchestrated by an asynchronous task submission system that dynamically allocates computational resources. Consequently, the architecture transforms the computational complexity, reducing the total time from a serial scenario, $T_{serial}=N_{files}\times N_{models}\times T_{fit}$ , to an optimized parallel equivalent, $T_{parallel}=max(\frac{N_{files}}{C_{files}},\frac{N_{models}}{C_{models}})\times T_{fit}$ . Furthermore, the multi-dimensional error evaluation process is also parallelized, ensuring that model selection occurs in real-time without introducing serial bottlenecks.

#### 2.2.3. Integration of Physical Constraints in the Optimization Framework

A fundamental principle of our methodology is the systematic and comprehensive integration of physical constraints throughout the entire analysis process. This design principle is of crucial importance, as it ensures that the final outputs are not only mathematically accurate, but also physically meaningful and directly interpretable. The present framework does not treat interpretability as a secondary consideration. Instead, it proactively leverages physical laws to guide algorithmic decision-making at three critical junctures: model selection, parameter optimization, and feature analysis. This approach overcomes the “black-box” nature that is characteristic of many data-driven methods.

During the preliminary model selection phase, the search space of the algorithm is inherently constrained. In lieu of exploring an abstract, infinite circuit topology space, the framework operates exclusively within a predefined library of candidate ECMs. This is illustrated in Supplementary Figure S2. The selection of models within this library is predicated on their proven theoretical and empirical validity in characterizing well-understood electrochemical phenomena, including electrode kinetics, mass transfer limitations, and the behavior of surface films or coatings. The initial model space constraint guarantees that any model ultimately selected by the algorithm is founded on a robust prior physical foundation, thereby anchoring the analysis in recognized electrochemical principles.

In the subsequent phase of parameter optimization, robust parameter space constraints are strictly enforced. The hybrid DE-LM algorithm does not perform unbounded searches; instead, exploration for each parameter value is strictly confined within the boundaries of physical reality. For instance, all passive elements, such as resistors, Warburg coefficients, and capacitors, are constrained to non-negative values. In a similar manner, the exponent n describing the constant phase element, which quantifies deviations from ideal capacitive behavior due to surface inhomogeneities or porosity, is confined to physically plausible ranges. These constraints are crucial for preventing convergence to mathematically optimal yet physically absurd solutions, a common pitfall in unconstrained nonlinear regression, thereby ensuring the physical integrity of the final fitted parameters.

This framework is intended to provide an interpretable feature space for downstream analytical tasks, such as concentration prediction, as demonstrated in the present applied research. The predictive model does not utilize raw, high-dimensional spectral data as input, which typically results in a “black-box” model. Instead, it employs a fitted, physically meaningful, low-dimensional set of circuit parameters as its input features. This critical design choice establishes a direct, transparent, and quantifiable link between a specific physical process—such as charge transfer—and the system’s macroscopic state, analogous to analyte concentration. This approach successfully overcomes the opacity that is often present in machine learning applications. The result is a model that is both highly accurate and directly interpretable within the established framework of electro-chemical principles.

## 3. Results and Discussion

### 3.1. Analysis of Data Sets

As illustrated in Table S1 of the supplemental information (SI), the analysis results of the constructed dataset were presented. The frequency dimension spans eight orders of magnitude (0.001–10^5^ Hz), designed to encompass the full-band response from quasi-static to high-frequency electromagnetic fields. The parameter distribution ranged from −0.015 to 319.430 for the real part and −58 to approximately 0 for the imaginary part, corresponding to the electromagnetic response characteristics from high-loss capacitive dielectrics to low-loss materials. Notably, duplicate and missing values in the dataset were uniformly set to 0.01, demonstrating the effectiveness of the adopted noise reduction method in balancing measurement noise and simulated ideal conditions.

A key strength of this dataset lies in its physically meaningful feature space. The weak correlation between the frequency and the real (−0.1758) and imaginary (0.1599) parts, coupled with the strong negative correlation between the real and imaginary parts themselves (−0.5545), accurately reflects the complex interdependencies between the real and imaginary impedance components, which are consistent with the Kramers–Kronig relations for a linearized electrochemical system [26].

The dataset’s richness is further evidenced by its statistical diversity. The frequency dimension analysis yielded a standard deviation of 1.44 × 10^3^ Hz, reflecting the significant variability of frequency-domain features. The real and imaginary parts exhibited standard deviations of 71.73 Ω and 9.86 Ω, respectively, effectively encompassing the entire dynamic range of the system from weak perturbations to significant responses.

Ultimately, the dataset demonstrates exceptional generalization capability, achieving a high generalizability score of 4.8 × 10^6^, which enables reliable extrapolation to similar scales. This superior performance stems from the implemented hybrid sampling strategy, which combines exponential sampling with physics-constrained adaptive sampling points. This strategy underscores the imperative for ensuring both authenticity and generalizability when integrating simulated data with real experimental data.

### 3.2. Optimization Analysis

#### 3.2.1. Validation of Error-Driven Optimization Efficacy and Multi-Model Parallel Fitting Results Through Multi-Threaded Implementation

**Figure 4:**
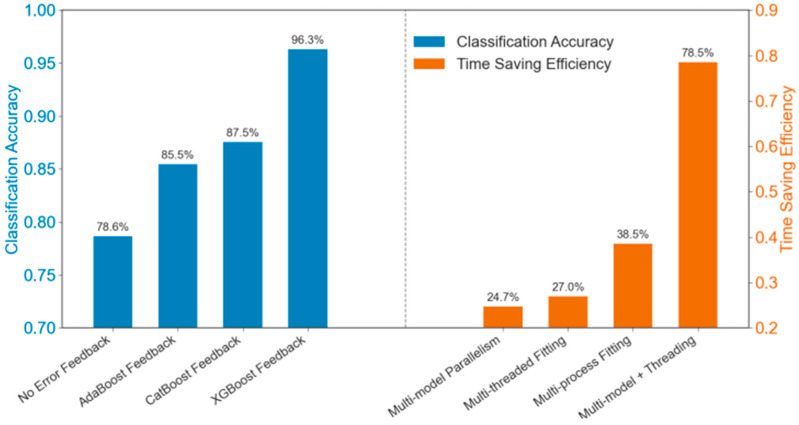
A comparative analysis of model optimization strategies across two performance dimensions.

The cross-sectional comparative analysis of the algorithms in the left-hand side of Figure 4 demonstrates that the implementation of XGBoost as an algorithm for error feedback adaptation exhibits a substantial performance advantage in the classification task. Assuming that without error feedback, the accuracy rate of the basic classifier was 78.65%. After introducing the error feedback mechanism of AdaBoost and CatBoost, the accuracy rate improved to 85.47% and 87.54% respectively. XGBoost further improved the accuracy to 96.32%, achieving an increase over the best traditional method (CatBoost) of about 8.78 percentage points. This performance improvement is primarily attributed to: (1) the regularized loss function implemented in XGBoost (combining L1 and L2 regularization), (2) the dynamic weight allocation strategy that optimizes gradients through second-derivative approximation. This results in a remarkable performance in terms of enhanced generalization ability and model stability. Preliminary experimental data demonstrate that XGBoost exhibits low error variance and performs effectively in feature adaptive processing, with automatic handling of missing values and feature combinations, and loss function optimization with support for customized regularization terms. These findings substantiate the conclusion that XGBoost provides a more efficient and stable solution to high-dimensional nonlinear classification problems.

The right-hand side of Figure 4 presents a comparative analysis of the systematic efficiency carried out for the three types of computational acceleration strategies: multi-model parallel fitting, multi-thread fitting, multi-process fitting, and their combined effects. The experimental data demonstrated that the composite parallel architecture exhibits a substantial advantage in terms of time-efficient optimization.

Among the single acceleration strategies, multi-process fitting (38.54% time reduction) demonstrated a more significant acceleration than multi-thread fitting (26.95%) and multi-model parallel fitting (24.72%). This is primarily attributed to the fact that process-level parallelism can circumvent the limitations of Python (Version 3.1)’s Global Interpreter Lock (GIL) in computationally intensive tasks and thus fully utilize multi-core CPU resources. Conversely, multithreading offers only modest efficiency gains in CPU-intensive scenarios due to the overhead associated with the GIL and thread switching. Moreover, multi-model parallel fitting was observed to exhibit a marginal degradation effect when implemented individually, a phenomenon attributed to potential resource competition and communication overhead among models.

The composite architecture’s impressive time reduction of 78.54% is attributable to the synergistic effect of the three strategies. Compared with the most effective single strategy (multi-process fitting), the composite architecture demonstrated a performance improvement of approximately 103.6%, evidencing the substantial advantages of compound acceleration effects in practical applications. It is important to acknowledge that while a theoretical prediction can be derived by summing the time reduction rates of the three single strategies to 90.21%, the actual composite acceleration effect was influenced by factors such as coordination among layers, task scheduling, and communication overhead, exhibiting a nonlinear characteristic rather than a simple linear superposition.

This hierarchical approach effectively assigns distinct roles: multi-process fitting achieves task partitioning across compute nodes at the macro level, multi-thread fitting optimizes I/O operations within a single process, and multi-model parallel fitting achieves parallel computation through inter-model task partitioning. Through this method, the system is able to drive a GTX 4060s graphics card (NVIDIA Corporation, Santa Clara, CA, USA) to up to 80% load by optimally setting the number of processes and threads.

#### 3.2.2. Performance Verification of the DE-LM Parameter Optimization Framework

As shown in Supplementary Table S2, the DE-LM hybrid optimization algorithm was applied to fit electrochemical impedance spectroscopy (EIS) parameters for selected models after implementing the XGBoost-based classification. The DE-LM algorithm demonstrates significant comprehensive advantages, as validated through comparative experiments. Regarding noise immunity, under data perturbation conditions from −10% to +10%, DE-LM achieved a stable interval coverage of 95–99%, a ~10% improvement over both the Differential Evolution (DE) and Levenberg–Marquardt (LM) algorithms individually. In parameter consistency analysis, the similarity score for DE-LM reached 0.9938, significantly outperforming DE (0.8587) and LM (0.8764), proving it effectively preserves the physical model’s constraints. Most notably, in error analysis, the DE-LM algorithm exhibited a fitting residual of −0.723, representing reductions of 48.6% and 31.9% compared to DE and LM methods, respectively.

The algorithm’s robustness was further scrutinized through a comprehensive sensitivity analysis, detailed in Figure S4a of the SI. From a total of 95,570 optimized initial parameters, even under single-parameter perturbations of ±10%, the DE-LM algorithm demonstrated significant parameter insensitivity. For less sensitive parameters (indices 0–4), similarity remained between 99.775% and 100%. Even for parameters with higher sensitivity (indices 5–9), the algorithm maintained an average baseline similarity of 95.23%, with the most sensitive parameter dropping only to 94.0%, validating its robustness across different parameter dimensions.

A multi-faceted validation of the optimization process itself was presented in Figure S4. The linear relationship between the initial and optimized parameters was validated via least squares linear regression (Figure S4b, R^2^ = 0.9999), confirming that the optimization did not introduce systematic bias. The Bland–Altman consistency validation (Figure S4c) demonstrated that the mean deviation between parameters was negligible (−0.015%), with 97.29% of data points falling within the consistency limits. Furthermore, the error convergence mechanism validation (Figure S4d) quantitatively showed a remarkable 72.3% reduction in the composite fitting error score. This unitless score, calculated from a weighted sum of six normalized metrics, was reduced from 1.99 (unoptimized) to 0.55 (optimized).

Therefore, the DE-LM algorithm demonstrated significant disturbance resistance, maintaining an average similarity of 97.84% (SD = 2.31) in a 10-dimensional parameter space, significantly outperforming traditional algorithms. The employment of a dual validation mechanism (R^2^ > 0.999 & LOA < 0.3%) and the proven error reduction effectiveness (Δε = 1.44) confirmed the algorithm’s optimized precision. This dual integration of global search and local optimization effectively overcome the trade-off between convergence accuracy and generalization capability faced with traditional single-mechanism algorithms while maintaining stability.

**Figure 5:**
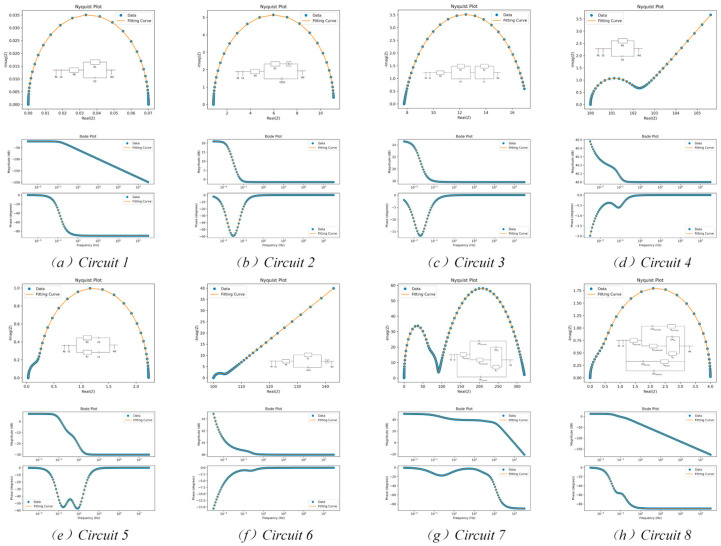
Example of fitting results for five solution-interface related equivalent circuits and three biological membrane equivalent models and evaluation indicators.

As illustrated in Figure 5, the results of the fitting process were presented for five solution-interface related equivalent circuits and three biofilm equivalent circuits. The original data points were represented by blue dots, and the impedance curves were represented by orange lines. The aforementioned curves were fitted using the predicted equivalent circuit model. The upper panel displays the Nyquist plot, while the lower panel presents the Bode plot. The Nyquist plot is a graphical representation that reveals the impedance characteristics of the system by displaying the real and imaginary parts of the complex impedance. For instance, the diameter of the semicircle is associated with the charge transfer resistance (R_ct_), which is indicative of the electrode reaction rate, while the gradient in the low-frequency region signifies diffusion-limited processes [27]. Conversely, the Bode plot demonstrates the relationship between impedance magnitude and phase as a function of frequency, thereby facilitating the identification of dynamic characteristics of disparate electrochemical processes and the extraction of features associated with charge transfer and diffusion through phase plots.

As demonstrated in Figure 5, the adaptive, multi-metric framework in this paper demonstrated a robust ability to differentiate between the candidate circuits, selecting the model whose fitted curve shows the closest alignment with the original data. The fundamental challenge in EIS is that distinct equivalent circuits can produce highly similar spectra. Conventional approaches relying on a single error metric are often unable to resolve this inherent ambiguity. To address this, our method employs dynamic weighting across six distinct error metrics—a strategy inspired by multi-criteria decision analysis [28]. This enables the detection of subtle yet critical deviations in the fitting quality, thereby offering a more robust and reliable criterion for model selection compared to traditional measures such as the residual sum of squares.

As demonstrated in Figure 5, our adaptive, multi-metric framework demonstrated a robust ability to differentiate between the candidate circuits, selecting the model whose fitted curve shows the closest alignment with the original data. We acknowledge the fundamental challenge that different circuits can yield similar spectra. Indeed, approaches relying on a single error metric often fail to resolve this ambiguity. However, by dynamically weighting six distinct error metrics, our method effectively captures subtle deviations in the fit, providing a more reliable criterion for model selection than a simple residual sum of squares.

It is evident that different equivalent circuit models produce distinct graphical features on the Nyquist plot that directly reflect the properties of the biofilm or electrode interface. For instance, the semicircle diameter quantifies charge transfer resistance, its suppression by a Constant Phase Element (CPE) indicates surface non-uniformity, and a low-frequency linear tail reveals diffusion limitations [29,30,31,32]. Consequently, a meticulous analysis of these features provides indispensable evidence for elucidating the structure, reaction kinetics, and transport mechanisms of the electrochemical interface.

**Figure 6:**
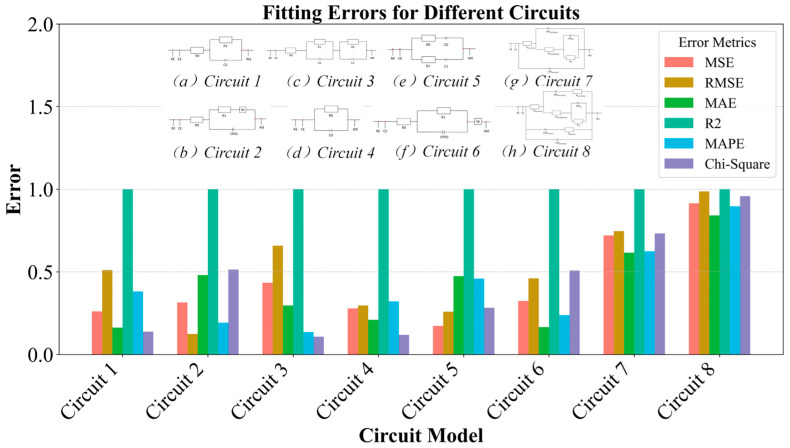
Quantitative comparison of fitting errors for the eight evaluated equivalent circuit models.

The experimental results demonstrate the method’s precise fitting capability across the full spectrum, accurately capturing the relationships between impedance magnitude, phase angle, and frequency. To transition from the qualitative assessment to a rigorous quantitative evaluation, a comprehensive error analysis was performed, with the results systematically presented in Figure 6. This figure details the fitting performance using five key statistical metrics: MSE, RMSE, MAE, MAPE and χ^2^ for each of the eight circuit models. Since the coefficient of determination (R^2^) consistently exceeded 0.99 across all cases, indicating excellent goodness-of-fit, it is not plotted alongside the error metrics. The quantitative analysis provides numerical validation and deeper insights supporting the conclusions in Figure 5.

The data in Figure 6, which displays the average final errors with standard deviation bars after DE-LM optimization for correctly classified spectra, quantitatively confirm the high quality of fit observed in our previous analysis. The Y-axis shows absolute metric values without normalization. As a result, the visual comparison between RMSE and MSE accurately reflects the mathematical relationship of the square root function, particularly for error values typically below 1, where RMSE is numerically larger than MSE.

A closer examination of the metrics reveals a clear and physically meaningful correlation between model complexity and fitting accuracy. As circuit models grow more intricate (e.g., progressing from simpler Circuits 1–6 to more complex Circuits 7 and 8), a slight increase in average error values and greater variability, which is evident from larger error bars, can be observed. This trend is anticipated due to the higher number of free parameters, which expands the dimensionality of the optimization search space and underscores the inherent trade-off between model complexity and fitting precision, even when using a robust optimization algorithm. Nonetheless, the overall fitting performance remains highly consistent across all models, affirming the effectiveness of our proposed framework.

In summary, the combined analysis of Figure 5 and Figure 6 offers a holistic evaluation of the model’s efficacy. While Figure 5 provides a clear qualitative confirmation of the model’s ability to replicate the impedance spectra, Figure 6 delivers the empirical, quantitative evidence of this accuracy. This dual-pronged analysis validates that the proposed methodology achieves both a high-fidelity representation of the electrochemical system’s behavior and quantitatively robust parameter estimations across a diverse range of circuit complexities.

### 3.3. Application Detection of BSA-CLB

**Figure 7:**
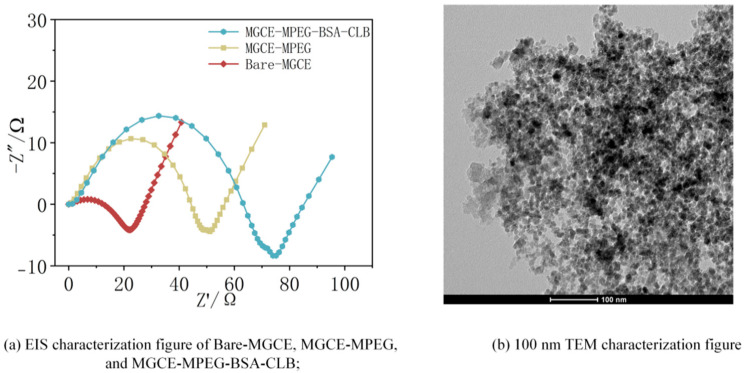
Characterization of the M-PEG-based electrochemical bio-sensing interface.

In brief, PEG-functionalized Fe_3_O_4_@SiO_2_ core–shell magnetic nanoparticles were synthesized via a multi-step co-precipitation and surface modification process [33,34]. Following their application for the adsorption of bovine serum albumin–clenbuterol (BSA-CLB) across a concentration gradient, the collected electrochemical impedance spectroscopy (EIS) data were subjected to model training and fitting. The raw EIS data and corresponding transmission electron microscopy (TEM) (USA)characterization of the nanoparticles were presented in Figure 7. The Nyquist plots in Figure 7a demonstrated a progressive increase in charge R_ct_ with each modification step—from the bare MGCE to the M-PEG layer and finally to the adsorbed BSA-CLB complex—confirming successful surface functionalization. Concurrently, the TEM image (Figure 7b) revealed uniformly dispersed nanoparticles, a morphology conducive to providing a high specific surface area for effective target adsorption.

**Figure 8:**
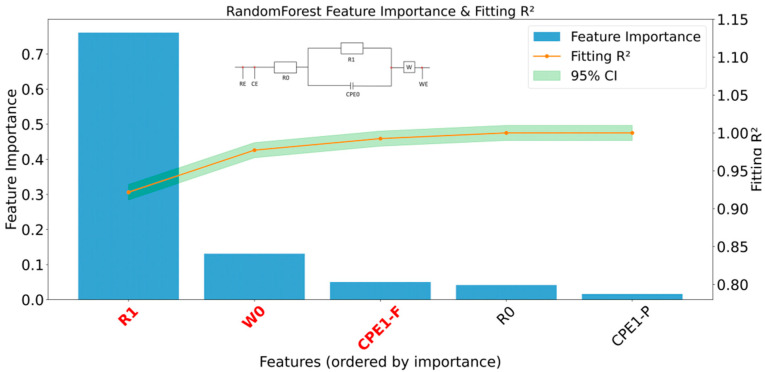
Feature importance analysis and predictive validation.

The analysis confirmed that the proposed classification and prediction model achieved optimal fitting (95.2% model selection accuracy) for biofilm applications with magnetic nanoparticle-deposited electrodes, as experimentally designed. Furthermore, subsequent parameter optimization using the DE-LM algorithm reduced fitting errors by 71.57%, demonstrating the effectiveness of global heuristic search. The specific data is shown in Figure S5 of the SI. For the purpose of correlation analysis, the circuit parameters were systematically decomposed: the features (X_1_–X_5_) were defined as the values of R_0_, CPE_1_-P, CPE_1_-F, R_1_, and W_0_ from the equivalent circuit model, while the target variable (Y) corresponded to the gradient concentration of BSA-CLB. The results of this correlation were shown in Figure 8.

It is crucial to distinguish the feature importance analysis in Figure 8 from the frequency-dependent behavior of circuit elements. Here, the objective is to predict a macroscopic variable (analyte concentration) from the entire impedance spectrum. To achieve this in an interpretable prospective, we first fit the spectrum to an ECM to extract a set of physically meaningful parameters [23]. These holistic, full-spectrum parameters (e.g., R_1_, W_0_) then serve as the “features” for the regression model. The analysis thus identifies which physical parameter, as a whole, is the most sensitive indicator of the change with the predicting results.

Figure 8 shows the feature importance and fitting performance of the RandomForest model, with the following ranking: R_1_ > W_0_ > CPE_1_-F > R_0_ > CPE_1_-P. This ranking quantitatively correlates key electrochemical processes with analyte concentrations.

Charge transfer resistance R_1_ (Its representative R_ct_) is the most significant feature, contributing approximately 73% of the predictive weight. This indicates that the reaction kinetics at the electrode interface are the most sensitive indicator to concentration changes, making R_ct_ a core parameter for quantitative analysis.

The Warburg impedance W_0_, representing mass transfer limitations, is the second most important (approximately 13%). The cumulative contribution of R_1_ and W_0_ exceeds 85%, indicating that the system is primarily controlled by the synergistic interaction of reaction kinetics and diffusion processes. The effects of the remaining parameters (CPE_1_-F, R_0_, CPE_1_-P) can be neglected.

The model’s fitting accuracy (Table 1) validates this importance analysis. To provide a direct benchmark, a quantitative comparison with expert manual fitting using ZView (Developed by Scribner Associates Inc. (Version 4.1b, Charlesville, VA, USA), is a software program for manually fitting EIS data) was conducted (detailed in Supplementary Tables S3 and S4). The parameters extracted by our automated algorithm showed excellent agreement with the manual fits, with relative errors for key parameters like R_1_ and W_0_ being only 0.19% and 0.41%, respectively. Furthermore, when used as features for concentration prediction, our algorithm’s parameters consistently outperformed those from manual fitting. When using only R_1_, R^2^ reached to 0.9218; when W_0_ added, R^2^ increased to 0.9772; and finally, after introducing CPE_1_-F and removing outliers, R^2^ reached 0.9925. This demonstrated that using the top 2–3 most relevant physical characteristics can achieve robust predictions.

In summary, this machine learning analysis established an interpretable mapping from electrochemical parameters to target concentrations. The model accurately identified R_1_ and diffusion W_0_ as the dominant factors in the system response. This method not only yielded a highly accurate predictive model but also enhanced its physical interpretability, validating the effectiveness of machine learning in analyzing complex impedance spectra and guiding sensor design.

To further validate the generality of our framework, we applied it to a dataset of bare structured electrode of varying surface areas with EIS measurements. The framework successfully identified the correct physical model with a 97.24% accuracy and yielded high-quality fits. More importantly, when using the extracted ECM parameters to predict the electrode area, the model achieved a predictive R^2^ of 0.99. The feature importance analysis confirmed that R_ct_ and a CPE parameter were the most significant predictors, which aligns perfectly with electrochemical theory. This confirms the framework’s versatility. The detailed results for this validation are provided in Figure S6 of the Supplementary Information.

## 4. Discussion

The proposed framework signifies a substantial advancement in the domains of automation and physically interpretable analysis of electrochemical impedance spectroscopy (EIS). The primary innovation of this study lies not in the individual components, but in the synergistic integration of a hybrid dataset with a multi-model, error-driven optimization mechanism that is rigorously guided by physical constraints at every stage. This integrated approach effectively mitigates the subjectivity and local-optimality pitfalls of traditional ECM fitting. A key advantage of this approach is its enhanced and reliable interpretability. By embedding physical constraints and correlating the outputs with electrochemical kinetics, it is possible to transcend the “black-box” paradigm and provide quantifiable, mechanistic insights into complex interfacial processes.

A critical aspect of this framework is its inherent ability to manage model complexity, a challenge traditionally addressed by statistical methods like the F-test. Our approach employs a competitive, multi-metric evaluation that intrinsically penalizes overfitting. To further validate our model selection, we incorporated an F-test as a post hoc verification step, which confirmed that in most cases, the model chosen by our framework represented a statistically significant improvement over simply nested alternatives. Unlike approaches focused exclusively on residual minimization, our framework employs a diverse set of metrics—including the χ^2^ test—to ensure that a more complex model is adopted only when it delivers comprehensive and meaningful improvement, both physically and statistically. This data-driven process serves as an intrinsic form of regularization, promoting model parsimony without a separate statistical test.

However, it is imperative to acknowledge the two significant limitations of this study. Firstly, the accuracy of the framework is sensitive to the representativeness of the initial hybrid dataset. Furthermore, significant differences between simulated and experimental data may introduce bias. Secondly, the prevailing physically based rules, which primarily target well-defined processes, may lack the requisite specificity to accurately model systems with highly coupled or non-ideal impedance responses.

In future research, more emphasis should be placed on enhancing the model’s adaptive capabilities, for instance, through the integration of active learning for intelligent data acquisition. Furthermore, the implementation of this interpretable framework in real-time monitoring of dynamic systems, including the tracking of catalyst degradation or the evolution of biofilms under in situ operating conditions, is a pivotal subsequent step to extend its practical value in complex electrochemical diagnostics.

## 5. Conclusions

Through systematic validation and multidimensional analysis, this study demonstrates that the proposed error-adaptive optimization framework achieves superior comprehensive performance in EIS analysis. The error feedback mechanism based on XGBoost improves classification accuracy from 78.65% in the baseline model to 96.32%, representing an 8.78 percentage point increase over the traditional optimal algorithm, CatBoost. The core of this improvement lies in the DE-LM hybrid algorithm, which combines global search and local optimization mechanisms. This approach maintains 95–99% robustness even when subjected to ±10% data perturbation, reduces fitted residuals by 48.6% and 31.9% compared to the DE and LM algorithms respectively, and improves parameter similarity to an exceptional 99.38%.

In terms of computational performance, the composite parallel architecture—characterized by its multi-process, multi-thread, and multi-model capabilities—achieved a total time efficiency optimization of 78.54%. The multi-process strategy was the most significant contributor, accounting for 38.54% of the observed acceleration effect, showcasing the architecture’s ability to effectively leverage modern multi-core processing ability.

Crucially, the framework successfully links algorithmic outputs to physical meaning. In the correlation analysis, charge transfer resistance R_1_ (=R_ct_) was found to be the dominant factor in the concentration prediction model, with a feature importance of 0.7. In contrast, W_0_ contributed 0.16, thereby quantitatively highlighting the pivotal roles of magnetic nanoparticle biofilm thickness and pore structure in detection sensitivity. Furthermore, the parameter mapping of CPE_1_-F/P confirmed the quantitative correlation between surface roughness (SD < 0.005σ) and changes in the Debye length of the double layer.

In conclusion, this study proposes a novel methodology for deriving an interpretable “structure-response-concentration” three-level mapping relationship for complex electrochemical systems. This approach is rigorously supported by quantitative validation of its error convergence mechanism (∆ε = 1.44) and the significant contribution of physical parameters to the predictive model (>85%), marking a significant step towards automated and physically meaningful EIS analysis.

## Figures and Tables

**Table 1:** Prediction results for different features.

Selected Features	R^2^
R_1	0.9218
R_1 + W_0	0.9772
R_1 + W_0 + CPE1_F	0.9925

## Data Availability

Data will be made available on request.

## Supplementary Materials

The following supporting information can be downloaded at: https://www.mdpi.com/article/10.3390/bios15090604/s1, Figure S1: Composition of the dataset; Figure S2: Overview and classification of Equivalent Circuit Models (ECMs) used in Electrochemical Impedance Spectroscopy (EIS); Figure S3: Flowchart of multi-model and multi-thread parallel fitting strategy; Figure S4: Comprehensive analysis of parameter robustness, consistency verification, and error optimization of the DE-LM collaborative optimization algorithm; Figure S5: Evaluation of the optimal equivalent circuit model and optimization of fitting error; Figure S6. Bare Electrode Data Classification, Errors and Predictions; Table S1: Data set feature analysis; Table S2: Comparison of parameter search algorithms; Table S3. Quantitative comparison of the algorithm with Zview fitting errors for samples of BSA-CLB data; Table S4. Prediction results for different features (circuit parameters obtained by the algorithm versus the distribution of circuit parameters obtained by zview fitting as a dataset).
