# Supplementary material for: Construction of an Electrochemical Impedance Spectroscopy Matching Method Based on Adaptive Multi-Error Driving and Application Testing for Biofilm Impedance Verification
Source: Biosensors (Basel). 2025 Sep 12;15(9):604. doi: 10.3390/bios15090604 (PMC12467955; doi:10.3390/bios15090604)
Supplement: Supplementary file 1 [file biosensors-15-00604-s001.zip › biosensors-3823774-supplementary.pdf]

Construction of an Electrochemical Impedance Spectroscopy Matching Method Based on Adaptive Multi-Error Driving and Application Testing for Biofilm Impedance Verification

Hanyang Bao [1], Fan Yu [1], Peiyan Dai [1], Boyu Guo [1] and Ying Xu [1,2,*]

[1] School of Automation, Hangzhou Dianzi University, Hangzhou 310027, China
[2] Provincial Key Laboratory of Soft Matter & Biomedical Materials, Wenzhou Institute of the University of Chinese Academy of Sciences (WIUCAS), Wenzhou 325000, China
* Correspondence: xuyingxy@hdu.edu.cn

The fundamental RC parallel circuit (Eq. S1) models the initial capacitive response of the double-layer formation and charge transfer at the electrode interface, characterized by a single semicircle in the Nyquist plot.

$$Z = Z_{eq} = \frac{Z_{R0} \times Z_{C0}}{Z_{R0} + Z_{C0}} = \frac{R0}{1 + j\omega C0R0} \tag{S1}$$

The dual-pathway model (Eq. S2) simulates systems with competitive interfacial phenomena, consisting of two parallel branches, where each branch contains a resistor and a capacitor in series.

$$Z = \frac{1}{\frac{1}{R_0 + \frac{1}{j\omega C_0}} + \frac{1}{R_1 + \frac{1}{j\omega C_1}}} \tag{S2}$$

The classical *Randles* circuit (Eq. S3) models kinetically-controlled reactions by combining a solution resistance (R0) with a parallel charge transfer resistance (R1) and double-layer capacitance (C0), where the semicircle diameter in the Nyquist plot directly quantifies the R1.

$$Z = Z_{R0} + Z_{eq} = R0 + \frac{R1}{1 + j\omega C0R1} \tag{S3}$$

The extended *Randles* circuit (Eq. S4) incorporates a Warburg element to model systems where both kinetic control and mass transport are significant. It consists of a solution resistance (R0) in series with an interface impedance, which separates the charge transfer semicircle from the low-frequency diffusion tail in the EIS plot.

$$Z(\omega) = R_0 + \frac{1}{\frac{1}{R_1 + Z_W(\omega)} + Q_0(j\omega)^{n_0}}, where Z_W(\omega) = \frac{\sigma_W}{\sqrt{\omega}}(1 - j) \tag{S4}$$

The dual time-constant model (Eq. S5) employs a series structure of two R-C parallel elements following a solution resistance (R0). This model is often used to simulate systems with multiple reaction interfaces, such as coated electrodes, which manifest as two distinct, overlapping arcs in the EIS plot.

$$Z(\omega) = R_0 + \frac{R_1}{1 + j\omega C_0 R_1} + \frac{R_2}{1 + j\omega C_1 R_2} \tag{S5}$$

The bare electrode model (Eq. S6) characterizes the fundamental interface using a solution resistance (R_sol), a polarization resistance (R_pol), and two Constant Phase Elements (CPEs) to represent the non-ideal double-layer capacitance and additional surface capacitances.

$$Z(\omega) = R_{SOLUTION} + \frac{1}{\sum_{i=1}^{3} Y_i} \text{ where } \begin{cases} Y_1 = Q_E (j\omega)^{n_E} \\ Y_2 = \dfrac{1}{R_P + \dfrac{R_{CT}}{1 + R_{CT} Q_{DL} (j\omega)^{n_{DL}}}} \\ Y_3 = Q_{EXC} (j\omega)^{n_{EXC}} \end{cases} \tag{S6}$$

The magnetic nano-biofilm model (Eq. S7) characterizes the modified interface by combining a solution resistance (R0), a parallel charge transfer unit (R1 // CPE0), and a Warburg element (W) in series to account for mass diffusion limitations through the film.

$$Z(\omega) = R_0 + \frac{R_1}{1 + R_1 Q_0 (j\omega)^{n_0}} + \frac{\sigma_W}{\sqrt{\omega}} (1 - j) \tag{S7}$$

The generalized biofilm model (Eq. S8) deconstructs the system into fundamental electrode components (R_ct, CPE_dl) and a dedicated parallel R_biofilm||C_biofilm element to specifically quantify the resistive and capacitive properties of the biofilm layer itself.

$$Z(\omega) = R_{SOLUTION} + \frac{1}{\sum_{i=1}^{4} Y_i} \text{ where } \begin{cases} Y_1 = \dfrac{1}{R_{ELECTRODE} + 1/(Q_E (j\omega)^{n_E})} \\ Y_2 = \dfrac{1}{R_{POLARIZATION} + \dfrac{R_{CT}}{1 + R_{CT} Q_{DL} (j\omega)^{n_{DL}}}} \\ Y_3 = \dfrac{1}{R_{BIOFILM} + 1/(Q_B (j\omega)^{n_B})} \\ Y_4 = Q_{EXC} (j\omega)^{n_{EXC}} \end{cases} \tag{S8}$$

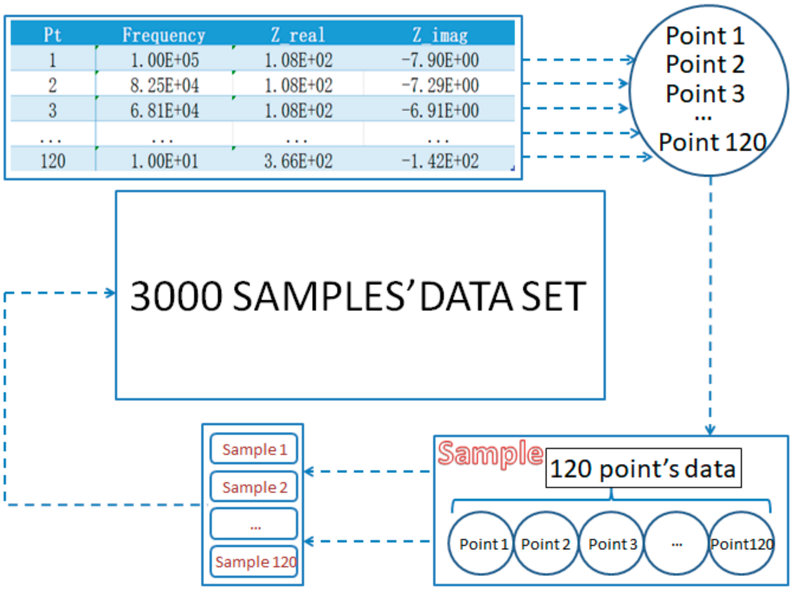

Figure S1. Composition of the dataset

Section S1.1: Methodology for Hybrid Dataset Construction

The hybrid dataset was constructed through a systematic, multi-stage process.

1. Simulated Data Generation:

To ensure both diversity and robustness, simulated data were generated using two

complementary strategies. First, Global Parameter Sampling was employed, where model parameters were drawn from a broad uniform distribution to create a wide variety of spectral shapes. Second, Local Parameter Perturbation was used to generate clusters of similar spectra by adding small, random perturbations to base parameter sets, mimicking experimental variability.

2. Data Preprocessing and Standardization:

Both experimental and simulated data underwent a unified preprocessing pipeline to ensure consistency. This pipeline included:

Noise Elimination: A median filter with a kernel size of 5 was applied to the real and imaginary parts of the impedance data. This non-linear filtering technique is effective at removing sporadic, high-frequency noise spikes without significantly distorting the underlying shape of the impedance curve.

Anomaly Detection: An Interquartile Range (IQR) based method was used to identify and remove outliers within each spectrum. Data points falling outside 1.5 times the IQR from the first and third quartiles were considered anomalies and were excluded. This statistical method was chosen for its robustness without assuming a specific data distribution.

Frequency Standardization: All spectra were resampled onto a common logarithmic frequency axis using cubic spline interpolation. This step was crucial for feature alignment, ensuring every sample possessed a consistent feature vector for the machine learning model.

This step was crucial for feature alignment, ensuring every sample possessed a consistent feature vector for the machine learning model.

The dataset comprises a total of 3000 samples distributed across eight distinct equivalent circuit model classes. The class distribution is mildly imbalanced: the five simpler specificity-related models each contain 300 samples (1500 total), while the three more complex biofilm models each contain 500 samples (1500 total). The ratio between the majority and minority classes is approximately 1.67:1. Given the mild nature of this imbalance and the inherent robustness of XGBoost to such distributional skew, we deemed that expliciting balancing techniques—such as oversampling (e.g., SMOTE)— was unnecessary. The high accuracy achieved validates this approach.

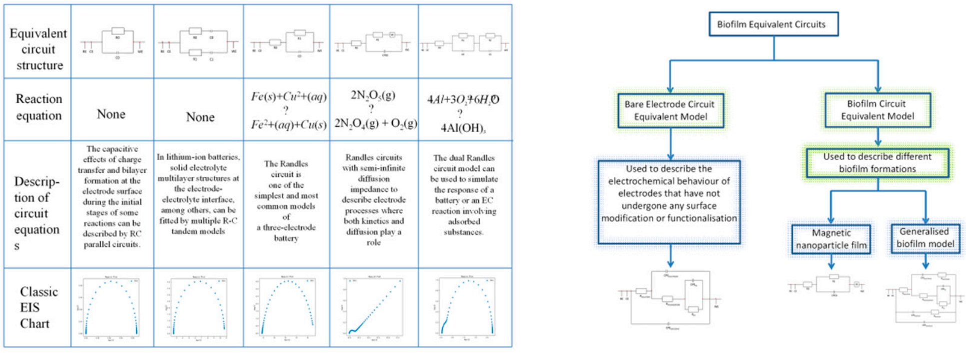

(a) RC&Randles Circuits 1-5

(b) Biofilm Circuits 6-8

Figure S2. Overview and classification of Equivalent Circuit Models (ECMs) used in Electrochemical Impedance Spectroscopy (EIS)

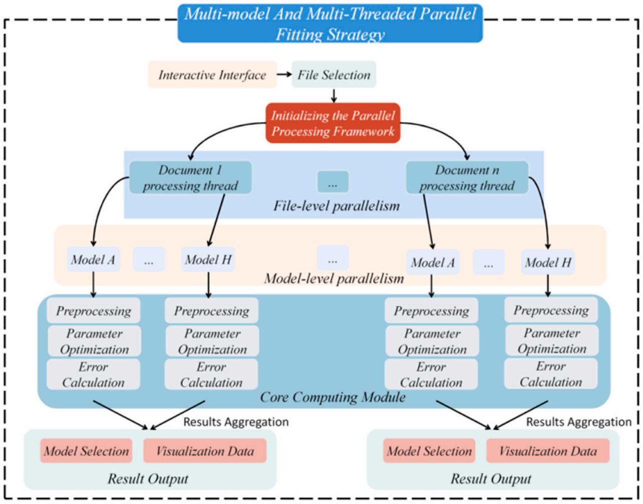

Figure S3. Flowchart of multi-model and multi-thread parallel fitting strategy

Table S1. Data set feature analysis

Analysis Dimension	Metric Name	Value/Range	Unit	Significance Statement
**Dynamic Range**	Frequency Span	[1.0×10^{-3}, 1.0×10^{5}]	Hz	Spanning eight orders of magnitude
	Real Permittivity (ε')	[-0.015, 319.430]	-	Includes negative permittivity phenomena
	Imaginary Permittivity (ε'')	[-58.000, -1.13×10^{-9}]	-	Characterizes strong loss up to nearly lossless materials
**Data Quality**	Redundancy Ratio	0.01%	-	Indicates near-complete elimination of data redundancy
	Missing Value Ratio	0.01%	-	Demonstrates near-complete, full-dimensional data coverage
**Feature Correlation**	Frequency–Real (Pearson r)	-0.1758	-	Corresponds to the non-linear nature of Kramers-Kronig relations
	Frequency–Imaginary	+0.1599	-	

	(Pearson r)			
	Real–Imaginary (Pearson r)	-0.5545	-	Corresponds to the prediction from Kramers-Kronig relations
**Generalization**	Frequency Standard Deviation (σ_f)	1.44×10^3	Hz	
**Ability**	Real Part Standard Deviation (σ_ε')	71.73	Ω	Reflects ultra-broadband frequency coverage
	Imaginary Part Standard Deviation (σ_ε'')	9.86	Ω	

Table S2. Comparison of parameter search algorithms

**Parameter search optimization methods**	**Robustness after disturbance (-10%-10%)**	**Similarity between initial and fitted parameters**	**BA 96% confidence interval fallout**	**Error Comparison**
Default Parameter	/	75.27%	73.81%	/
DE	83-89%	85.87%	83.67%	-48.62%
LM	85-91%	87.64%	84.24%	-54.80%
**DE-LM**	**95-99%**	**99.38%**	**97.29%**	**-72.30%**

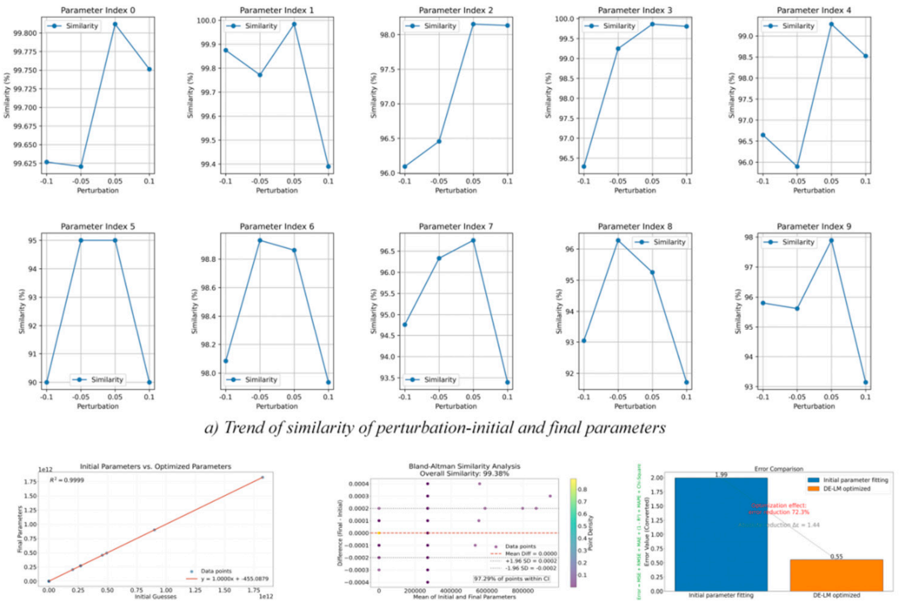

a) Trend of similarity of perturbation-initial and final parameters

b) Initial-final parameter linear fit *c) Bland-Altman similarity analysis* *d) Error results*

Figure S4. Comprehensive analysis of parameter robustness, consistency verification, and error optimization of the DE-LM collaborative optimization algorithm

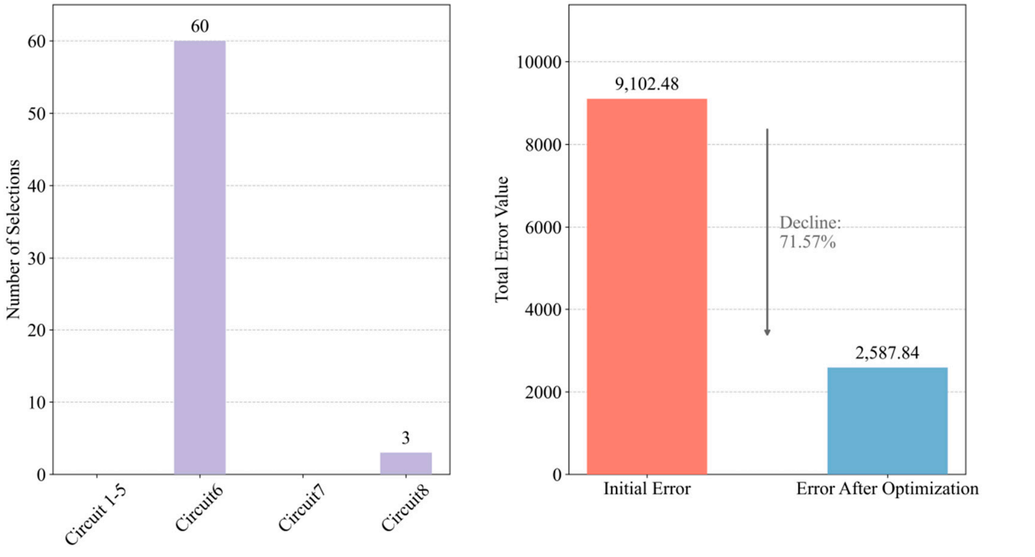

(A) Analysis of Circuit Selections (B) Error Comparison(Sum of six error parameters in 63 groups)

Figure S5. Evaluation of the optimal equivalent circuit model and optimization of fitting error.

Table S3. Quantitative comparison of the algorithm with Zview fitting errors for samples of BSA-CLB data

Error Type	Z-View vs Algorithm
Overall R_0 Error	0.52%
Overall CPE_1-P Error	1.08%
Overall CPE_1-F Error	0.67%
Overall R_1 Error	0.19%
Overall W0 Error	0.41%
Overall Chi-square	0.52%

Table S4. Prediction results for different features (circuit parameters obtained by the algorithm versus the distribution of circuit parameters obtained by zview fitting as a dataset)

Selected Features	Zview_R^2	Algorithm_ R^2
R_1	0.9157	0.9218
R_1+W_0	0.9769	0.9772
R_1+W_0+CPE1_F	0.9866	0.9925

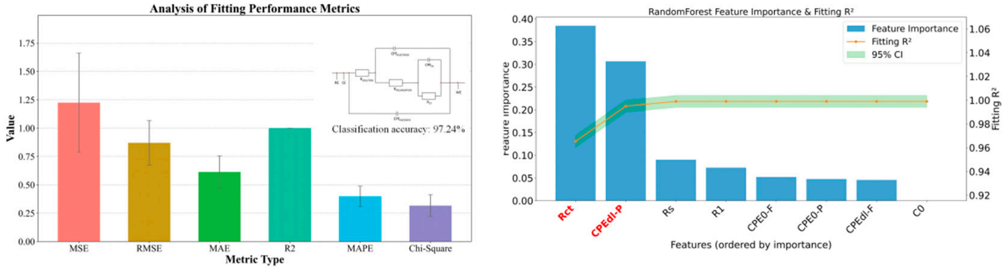

a) Classification and error of the fit to bare electrode data

b) Forecasts with electrode area as the forecasting target

Figure S6. Bare Electrode Data Classification, Errors and Predictions
